# A novel robotic surgical assistant for total knee arthroplasty has a learning curve ranging from 6 to 14 cases and exhibits high accuracy in tibial bone cuts

**DOI:** 10.1186/s13018-024-04984-6

**Published:** 2024-08-17

**Authors:** Nimit Thongpulsawad, Chaiwat Achawakulthep, Tawan Intiyanaravut

**Affiliations:** Department of Orthopedic Surgery, Golden Jubilee Medical Center, Faculty of Medicine Siriraj Hospital, Mahidol University, Nakhon Pathom, Thailand

**Keywords:** Accuracy, Cumulative summation analysis, Learning curve, Operative time, Robot-assisted system, ROSA knee system, Total knee arthroplasty

## Abstract

**Background:**

The adoption of robot-assisted total knee arthroplasty (TKA) aims to enhance the precision of implant positioning and limb alignment. Despite its benefits, the adoption of such technology is often accompanied by an initial learning curve, which may result in increased operative times. This study sought to determine the learning curve for the ROSA (Robotic Surgical Assistant) Knee System (Zimmer Biomet) in performing TKA and to evaluate the accuracy of the system in executing bone cuts and angles as planned. The hypothesis of this study was that cumulative experience with this robotic system would lead to reduced operative times. Additionally, the ROSA system demonstrated reliability in terms of the accuracy and reproducibility of bone cuts.

**Methods:**

In this retrospective observational study, we examined 110 medical records from 95 patients who underwent ROSA-assisted TKA performed by three surgeons. We employed the cumulative summation methodology to assess the learning curves related to operative time. Furthermore, we evaluated the accuracy of the ROSA Knee System in performing TKA by comparing planned versus validated values for femoral and tibial bone cuts and angles.

**Results:**

The learning curve for the ROSA Knee System spanned 14, 14, and 6 cases for the respective surgeons, with operative times decreasing by 22 min upon reaching proficiency (70.8 vs. 48.9 min; *p* < 0.001). Significant discrepancies were observed between the average planned and validated cuts and angles for femoral bone cuts (0.4 degree *±* 2.4 for femoral flexion, 0.1 degree *±* 0.6 for femoral coronal alignment, 0.3 mm *±* 1.2 for distal medial femoral resection, 1.4 mm *±* 8.8 for distal lateral femoral resection) and hip–knee–ankle axis alignment (0.3 degree *±* 1.9 )(*p* < 0.05) but not for tibial bone cuts. Differences between planned and validated measurements during the learning and proficiency phases were nonsignificant across all parameters, except for the femoral flexion angle (0.42 degree *±* 0.8 vs. 0.44 degree *±* 2.7) (*p* = 0.49).

**Conclusion:**

The ROSA Knee System can be integrated into surgical workflows after a modest learning curve of 6 to 14 cases. The system demonstrated high accuracy and reproducibility, particularly for tibial bone cuts. Acknowledging the learning curve associated with new robot-assisted TKA technologies is vital for their effective implementation.

## Introduction

Total knee arthroplasty (TKA) is deemed an effective intervention for advanced-stage osteoarthritis, with ongoing advancements in implant design, prosthetic materials, and surgical techniques aimed at enhancing TKA longevity and patient satisfaction. Despite these advancements, reports suggest that up to 20% of TKA recipients remain dissatisfied [1–6]. The pursuit of improved surgical precision, patient satisfaction, and functional outcomes has propelled the development of new technologies in TKA, notably robot-assisted TKA. This approach has shown promise in enhancing the accuracy of implant positioning and limb alignment compared to traditional jig-based TKA [5, 7]. Nonetheless, the introduction of novel techniques often entails a learning curve, typically associated with extended operative times and a heightened risk of adverse events [8, 9]. At present, the orthopedic field boasts a variety of robotic systems that are classified into active systems, semi-active systems, and passive systems. These classifications are based on the surgeon’s required interaction level during resection [10]. Among these systems, the ROSA (Robotic Surgical Assistant) Knee System (Zimmer Biomet, Warsaw, Indiana, USA) is categorized as a passive robotic system [11]. In this setup, the surgeon directs the procedure while leveraging a sophisticated robotic tool for surgical execution. The advantage of this system is that surgeons do not have to alter their operating techniques as extensively as with active or semi-active systems, resulting in lower effort required for adopting the ROSA system. This study aimed to delineate the learning curve associated with using the ROSA Knee System to perform TKA and to evaluate the system’s precision in performing planned bone cuts and angles. Additionally, this study sought to assess the reproducibility of bone cuts facilitated by this innovative technology. We hypothesized that cumulative experience with this novel robotic system would result in reduced operative times. Furthermore, the accuracy and reproducibility of the ROSA system in performing bone cuts are reliable.

## Methods

This retrospective observational study was conducted through a review of medical records from patients treated at the Golden Jubilee Medical Center, Mahidol University, Nakhon Pathom, Thailand. The research received ethical approval from the Institutional Review Board of Siriraj Hospital, Mahidol University, under protocol number 169/2566 (IRB3). The dataset comprised 110 medical records from 95 patients who underwent robotic-assisted total knee arthroplasty (RA TKA) between December 2021 and December 2022. Three surgeons, all certified in robotic-arm assisted ROSA Knee System platform (Zimmer Biomet), performed the operations. The surgeries involved the use of cemented, fixed-bearing, and posterior-stabilized knee prostheses (Persona, NexGen; LPS, LPS-flex, Gender Solutions). Preoperative long-standing radiographs, image-based technique, was done in all patient.

### Surgical techniques

**Fig. 1:**
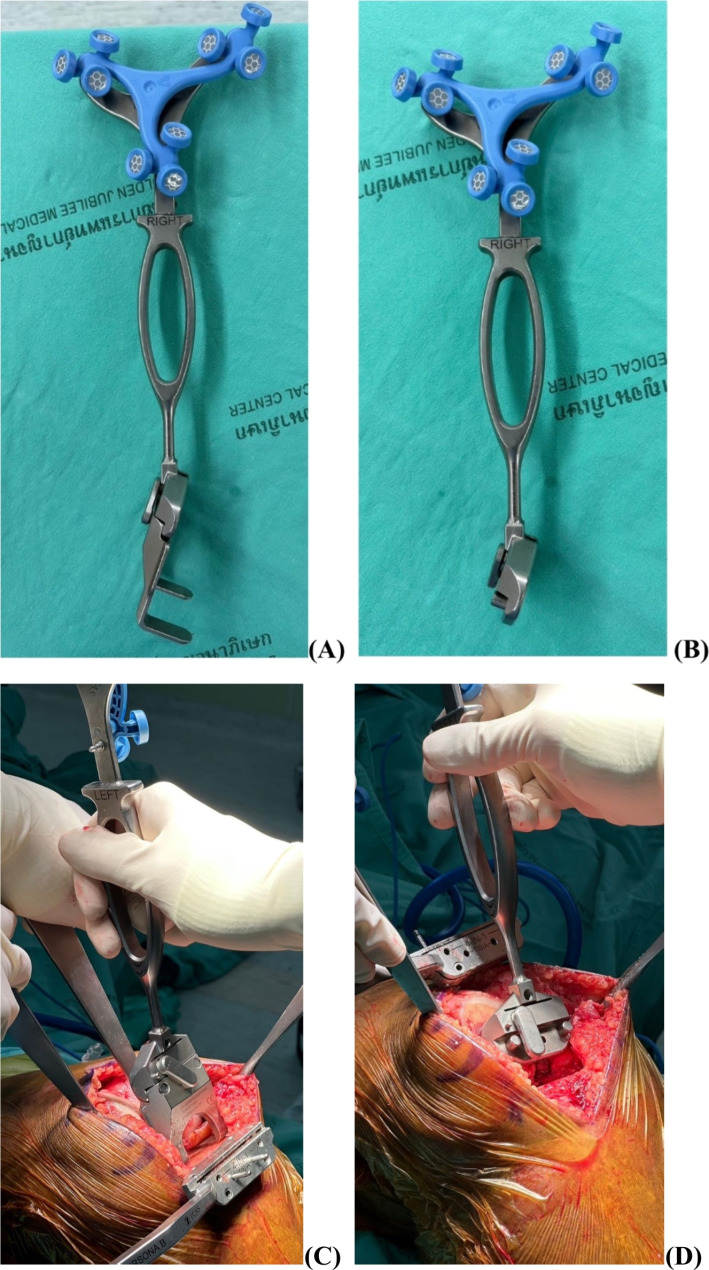
Rosa knee tibia and femur validation tools (**A, B**) and intraoperative validation processes for tibia and femur (**C, D**)

The operative procedure entailed the insertion of bicortical diaphyseal array reference pins into the distal femur and proximal tibia. A medial parapatellar approach was used in all patients. Bone registration, which is crucial for surgical navigation, followed the established ROSA Knee System protocol. This protocol required the identification and registration of several anatomical landmarks: the femoral head center, femoral canal entry point, trochlear groove, anterior cortex, medial and lateral epicondyles, distal and posterior femoral condyles, malleoli, medial and lateral tibial plateau, tibial canal entry, posterior cruciate ligament insertion, and tibial tuberosity. After the anatomical landmark registration process, surgical planning and joint balancing were performed. The position of implants, thickness, and angle degrees of bone cuts were adjusted until the desired positions were achieved. Next is the bone cutting process, which is performed manually by surgeons with a saw blade through the cutting slots of the robotic arm. The ROSA validation tools version 1.2 were used to evaluate bone cuts by placing the tools on the flat surfaces of bone cuts (Fig. 1). After all trial components and final implants were assembled, the evaluation process was conducted, and the time taken in each step was recorded.

The inclusion criteria for this investigation were patients aged 50 years or older who were diagnosed with knee osteoarthritis and underwent RA TKA. Patients whose procedures transitioned from RA TKA to conventional methods and those whose procedures were not performed by a full-time arthroplasty surgeon at the Golden Jubilee Medical Center were excluded.

Patient demographic and clinical data, including age, sex, body mass index, American Society of Anesthesiologists physical status classification, and Oxford Knee Score, were collected retrospectively.

### Outcome measurements

#### Operative time

The definition of total robotic operative time encompassed the duration of robotic unit use, incorporating the surgical approach, bone landmarking, planning, bone cutting, and evaluation phases.

#### Cumulative summation analysis

Learning curves regarding operative time were scrutinized using the cumulative summation (CUSUM) method, as previously described [7, 12]. The CUSUM analyses were performed using the aggregate mean operative times of the RA TKA group, with CUSUM values reflecting the cumulative disparity between individual data points and the group’s overall mean. An inflection in the CUSUM trend indicated a shift from the learning phase to proficiency.

#### Accuracy outcome

This study compared planned versus validated bone cuts and angles using the ROSA Knee System’s validation tools. The mean discrepancies between the planned and validated cuts during both the learning and proficiency phases were analyzed. This study also examined the differences between preoperative plans and final prosthetic component sizes installed during surgery.

### Statistical analysis

Descriptive statistics, including means and standard deviations, were computed for all parameters (cuts and angles) that were planned and validated using the ROSA system. The average error for each paired sample was ascertained by calculating the mean of the differences between matched data for each patient. Subsequently, a dedicated t test was conducted. A 95% confidence interval was established for the t test, and statistical significance was predetermined at *p* < 0.05 for all tests. All the statistical analyses were conducted with IBM SPSS Statistics, version 25 (IBM Corp, Armonk, NY, USA).

## Results

### Learning curve outcome (operative time)

**Fig. 2:**
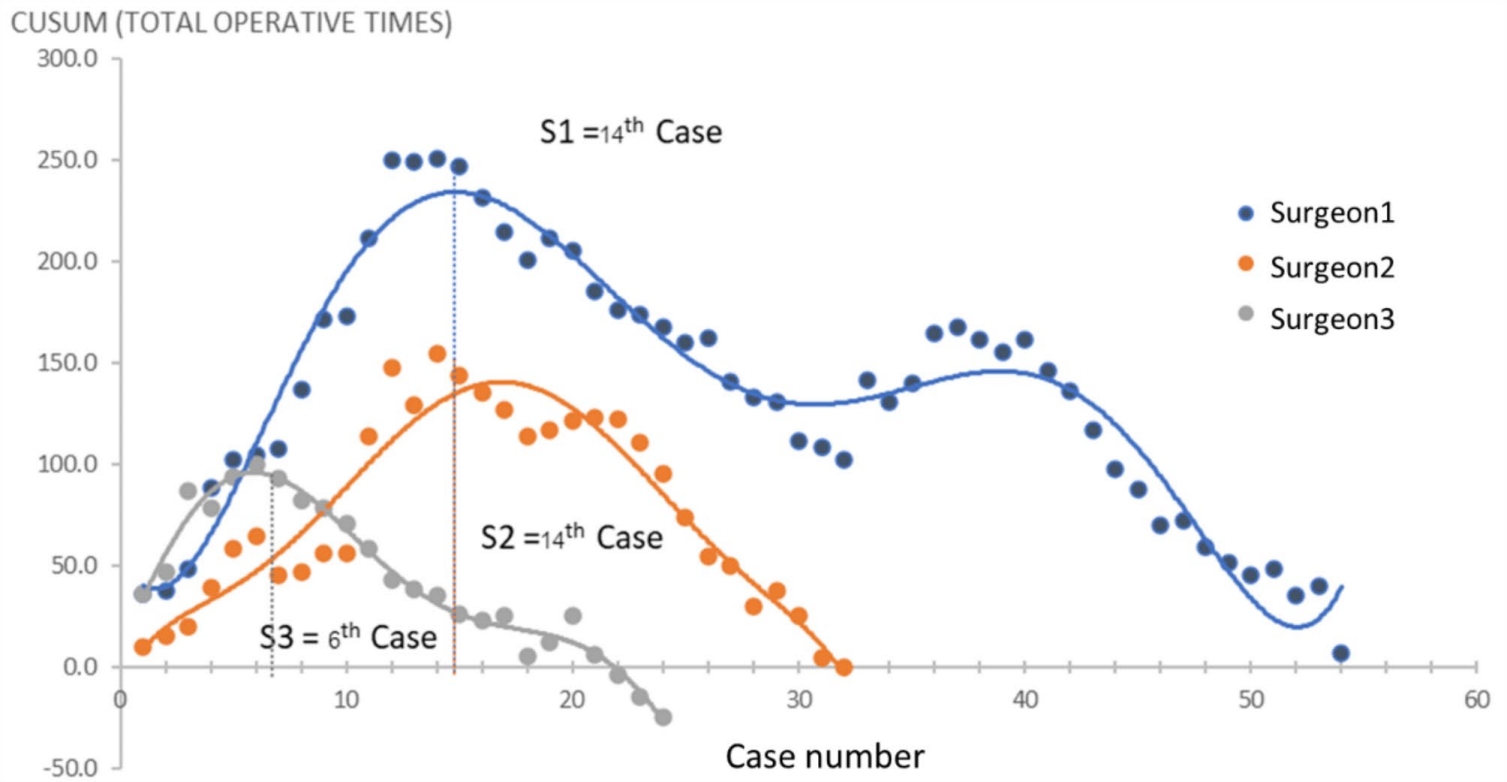
Cumulative summation analysis depicting the learning curves (operative duration) in robotic-arm assisted TKA among three experienced surgeons. The inflection point, highlighted by the vertical dotted line, delineates the transition from the learning phase to the proficiency phase

Throughout the study duration, the three surgeons completed from 24 to 54 RA TKAs each. The individual inflection point of the learning curve for each surgeon occurred at the 14th, 14th, and 6th case (Fig. 2), indicating the transition from the learning phase to proficiency. Analysis of baseline patient demographic data revealed no significant differences between the learning and proficiency phases regarding the age, preoperative knee functional score, or body mass index of patients. However, during the learning phase, there were significantly greater percentages of patients who were male and patients who were classified as American Society of Anesthesiologists class 2 than during the proficiency phase (*p* < 0.01 and *p* < 0.05, respectively). The demographic and clinical data of the patients are detailed in Table 1.

**Table 1:** Patient demographic and clinical characteristics in the learning and proficiency phases

	Learning phase	Proficiency phase	*p*
Male sex (%)	15 (13.6%)	10 (9.1%)	< 0.001*
ASA class 2 (%)	16 (15%)	17 (15.9%)	< 0.05*
ASA class 3 (%)	17 (15.9%)	57 (53.3%)	
Age (year ± SD)	71.2 ± 6.8	71.8 ± 7.5	ns
BMI (kg/m^2^ ± SD)	28.4 ± 4.0	27.4 ± 4.0	ns
Oxford Knee Score	22.2 ± 9.0	22.2 ± 9.0	ns

BMI, body mass index; ns, not significant; SD, standard deviation

The total operative time during the learning phase exceeded that of the proficiency phase by 22 min (70.8 vs. 48.9 min; *p* < 0.001). This discrepancy was attributed to prolonged durations in the surgical approach and bone registration (1 min longer; *p* < 0.05), surgical planning and balancing (20 min longer; *p* < 0.001), and bone preparation and component trialing (3 min longer; *p* < 0.05). Nonetheless, the duration of the surgical evaluation did not significantly differ between the learning and proficiency phases (Table 2).

**Table 2:** Comparison of operative duration across surgical stages between learning and proficiency phases

Surgical stage (min) ± SD	Learning phase	Proficiency phase	*p*
Surgical approach and bone registration	6.1 ± 2.6	4.8 ± 3.1	< 0.05
Surgical planning and joint balancing	33.9 ± 16.7	13.3 ± 9.5	< 0.001
Bone preparation and component trialing	17.8 ± 8.8	14.1 ± 5.7	< 0.05
Surgical evaluation	15.1 ± 9.5	17.1 ± 11.3	ns
Total operative time	72.8 ± 20.8	49.2 ± 15.6	< 0.001

ns, not significant; SD, standard deviation

### Accuracy outcome

Analysis of the average planned versus validated measurements—covering femoral and tibial cuts, femoral flexion and axis, tibial slope and axis—using the ROSA Knee System was conducted. This assessment revealed statistically significant discrepancies in the angles for femoral flexion, femoral coronal alignment, and overall limb alignment. Additionally, notable differences emerged in the cut thicknesses for distal medial and lateral femoral resection (Table 3). Conversely, tibial bone cuts displayed no significant differences. The differences spanned from 0.32 mm for distal medial femoral resection up to 1.59 mm for proximal lateral tibial resection, with standard deviations ranging from 1.2 to 11.7. For the cut angles, the disparities between the planned and achieved angles for both the femur (flexion and coronal axis) and the tibia (slope and coronal axis) varied from a minimum of 0.1 degrees in femoral coronal alignment to 1.4 degrees in femoral flexion, with standard deviations of 0.6 to 2.4.

**Table 3:** Comparative analysis of planned versus validated bone resections and angular measurements

Bone cuts and angles	Planned value	Validated value	Difference	*p*
Femoral flexion (deg)	3.0 ± 0.1	2.6 ± 2.4	0.4 ± 2.4	< 0.001
Femoral coronal alignment (deg)	-0.4* ± 0.7	-0.5* ± 0.9	0.1 ± 0.6	< 0.05
Distal medial femoral resection (mm)	8.8 ± 1.7	8.4 ± 1.6	0.3 ± 1.2	< 0.05
Distal lateral femoral resection (mm)	9.3 ± 9.0	8.0 ± 1.6	1.4 ± 8.8	< 0.001
Tibial slope (deg)	4.1 ± 1.1	4.3 ± 2.0	-0.2 ± 1.7	ns
Tibial coronal alignment (deg)	-0.4* ± 0.7	-0.2* ± 1.0	-0.2 ± 0.7	ns
Proximal medial tibial resection (mm)	3.9 ± 2.1	4.4 ± 4.6	-0.5 ± 4.1	ns
Proximal lateral tibial resection (mm)	9.8 ± 10.3	8.2 ± 5.2	1.6 ± 11.7	ns
Overall limb alignment (deg)	-0.9* ± 1.2	-1.1* ± 2.2	0.3 ± 1.9	< 0.05

* Minus values represent varus angle

ns, not significant

The average differences between the planned and validated measurements for each phase are detailed in Table 4. There were no significant disparities between the learning and proficiency phases across all bone cuts, with the exception of femoral flexion.

**Table 4:** Mean discrepancies in bone resection and angular measurements between learning and proficiency phases

Average difference ± SD	Learning phase	Proficiency phase	*p*
Femoral flexion (deg)	0.42 ± 0.8	0.44 ± 2.7	0.49
Femoral coronal alignment (deg)	− 0.05 ± 0.9	0.16 ± 0.5	ns
Distal medial femoral resection (mm)	0.50 ± 0.8	0.25 ± 1.3	ns
Distal lateral femoral resection (mm)	0.41 ± 0.9	1.69 ± 10.2	ns
Tibial slope (deg)	-0.28 ± 1.7	-0.14 ± 1.7	ns
Tibial coronal alignment (deg)	-0.24 ± 0.8	-0.13 ± 0.7	ns
Proximal medial tibial resection (mm)	0.05 ± 1.1	-0.64 ± 4.7	ns
Proximal lateral tibial resection (mm)	-0.19 ± 1.2	2.15 ± 13.4	ns
Overall limb alignment (deg)	0.53 ± 3.3	0.18 ± 1.0	ns

ns, not significant; SD, standard deviation

**Fig. 3:**
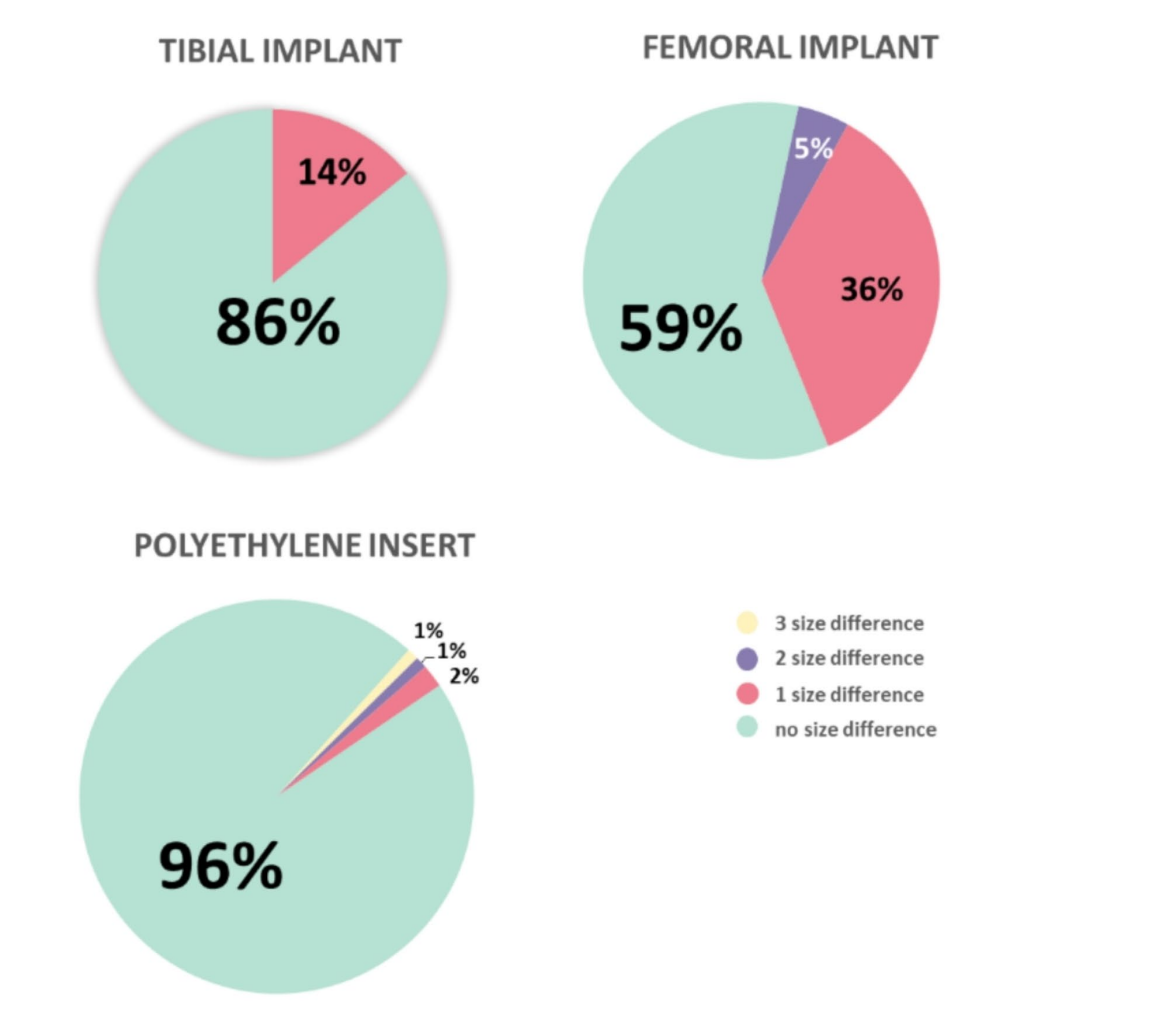
Accuracy of implant component size selection: planned versus actual outcomes

A comparison between planned and actual implant sizes demonstrated 86% accuracy for tibial implants, 59% for femoral implants, and 96% for polyethylene inserts (Fig. 3). Deviations of more than one size were recorded in 5% of femoral implants and 2% of polyethylene inserts, while tibial implants did not deviate by more than one size in any case. The analysis indicated no discernible learning curve in the accuracy of component planning.

## Discussion

This study’s primary finding was that the ROSA Knee System exhibited a learning curve ranging from 6 to 14 patients. Comparable findings were noted in prior investigations reporting on the learning curve of the ROSA Knee System, with operative times for three surgeons indicating a learning curve spanning 6 to 11 cases [13] and 5 to 15 cases, [14] using CUSUM analysis. These values align closely with our observations. Research on other optically guided RA systems, such as the Mako RIO (Stryker, Kalamazoo, MI, USA), [7, 15, 16] also revealed similar learning curves regarding operative time, notwithstanding design variations. Earlier evaluations have documented learning curves for diverse RA TKA systems ranging widely from 6 to 43 patients [7, 13, 15–19]. Upon reaching proficiency in performing TKA using the ROSA Knee System, we observed a reduction in operative time of an average of 22 min, primarily during the surgical planning and balancing stage. Tay et al. [19]. identified a learning curve of 16 cases and an increased operative time of 12 min during the learning phase. However, their findings indicated that this increase occurred in the surgical approach, bone registration, and bone preparation stages, which contrasts with our study’s results.

Another noteworthy outcome of this investigation is the high accuracy of the ROSA Knee System in executing planned cuts and angles, particularly regarding the tibial cut. The discrepancy between the thickness of the cuts planned and verified with the robotic validation tool can be rationalized considering the inherent thickness of the saw blade employed in traditional bone cutting methodologies. Our findings suggest that tibial cuts are executed with greater accuracy than femoral cuts. This difference may stem from the directional nature of the saw during tibial bone cuts, which are performed in the horizontal plane and are therefore simpler to control than the vertical plane maneuvers required for femoral bone cuts.

When assessing the precision of bone cuts across the learning and proficiency phases, our analysis revealed no notable difference for the cuts except for femoral flexion. This underscores the high reproducibility of the ROSA Knee System, particularly for tibial bone cuts. Our findings are in line with those reported by Sires et al., [20] who investigated the Mako total knee robotic arm-assisted surgery system (Stryker) across 37 consecutive TKAs. They quantified the mean discrepancies between the planned and actual measurements using a navigated probe system, identifying a mean deviation of 0.38 mm for distal femoral cuts, 0.46° in the coronal angle, and 0.55° in flexion for femoral components. For tibial cuts, a mean difference of 0.37 mm was reported, along with variations of 0.53° in the coronal angle and 0.59° in the slope. Similarly, Parratte et al. [21]. conducted a study utilizing the ROSA Knee System (Zimmer Biomet) on 30 TKAs performed on 15 frozen cadaveric specimens. They reported that femoral and tibial coronal and sagittal alignment errors exhibited standard deviations of less than 1, measured using the Optical Navigation System (ORTHOsoft, Zimmer Biomet).

Regarding hip–knee–ankle (HKA) axis alignment, the mean discrepancy between the planned and validated angles in our study was 0.3 degrees, with a standard deviation of 1.9 (*p* = 0.19). The planned HKA axis exhibited an average varus of 0.9 degrees (SD = 1.2), while the achieved angle averaged a varus of 1.1 degrees (SD = 2.2). Although this difference reached statistical significance, its clinical relevance is questionable. It is well documented that restoring the mechanical axis within 3 degrees of neutral correlates with better clinical outcomes and increased prosthesis longevity [22, 23]. Our analysis identified eight patients (5%) in which the HKA axis deviated from these optimal parameters. Nevertheless, all patients showed an improvement in Oxford Knee Scores 3 months after their operations. Our approach diverges from strict mechanical alignment, advocating instead for the concept of functional axis alignment. Notably, one patient experienced extreme varus of 12 degrees measured intraoperatively using the robotic tool, which contrasted with postoperative long-standing films revealing 5 degrees of varus. This discrepancy might stem from an error associated with the reference pin. The reliability of bone cuts, angles, and HKA axis evaluations conducted with robotic tools might be susceptible to errors. These inaccuracies can arise from various factors, such as the motion of reference pins, the roughness of bone cut surfaces, soft tissue obstructing the validation tool and bone cuts, or errors by the operator in not accurately positioning the validation tool on the bone cuts. In their investigation, Figueroa et al. [24]. evaluated the precision of a robotic-arm system for replicating planned bone cuts. Their investigation drew upon 173 TKAs and employed an imageless passive robotic system. By contrasting intraoperative recorded cuts with postoperative computed tomography scans for each patient, they found a mean disparity for HKA-predicted versus HKA-measured of 0.4° ± 2.4° toward more varus, with 83% of HKA angles remaining within a 3-degree variance.

Our study achieved component planning accuracy rates of 59% for the femoral component, 86% for the tibial component, and 96% for the polyethylene insert using the ROSA Knee System. Actual implant sizes deviated from preoperative plans by more than two sizes in only 7% of patients, predominantly concerning the femoral component (5%) and the polyethylene insert (2%). No such deviations were observed for the tibial component. There were no discernible differences in component size accuracy between the learning and proficiency phases. Notably, the tibial component exhibited the highest precision in size planning because it does not influence ligament balancing. Conversely, adjustments in the sizes of the femoral component and polyethylene inserts impact gap balancing.

This study has several limitations that warrant mention. First, no comparison was made between the operative times of RA TKA and those of conventional TKA methodologies. Instead, our investigation concentrated solely on the operative time within the RA-TKA cohort and the diminution of time upon attaining proficiency. Second, we did not conduct any assessment of clinical outcomes for the patients included in our study. Third, the validity of the accuracy verification for both the thickness of bone cuts and the angles is questionable. This is because our methodology relied exclusively on robotic validation tools without caliper confirmation of the actual thickness of the bone pieces or the use of postoperative imaging to verify the cut angles. Last, as our institution is a university hospital that frequently engages in educational demonstrations within the operative setting, this aspect may have influenced operative times in certain cases.

## Conclusions

The implementation of the ROSA Knee System for performing TKA incurred a learning curve spanning 6 to 14 patients. Proficiency in using the system resulted in a substantial reduction of 22 min in the operative time, primarily during the surgical planning and balancing phase. This robotic-assisted TKA system demonstrates superior accuracy and reproducibility, especially for tibial bone cuts and sizing of the tibial implant. These findings underscore the ability of the ROSA Knee System to seamlessly integrate into surgical workflows with a minimal learning curve. However, acknowledging the learning curve associated with the introduction of novel robotic-assisted TKA systems is essential for comprehending the broader implications of integrating this technology into orthopedic surgical practices.

## Data Availability

No datasets were generated or analysed during the current study.
